# An accelerating wind tunnel for testing untethered bodies in transverse gusts

**DOI:** 10.1007/s00348-025-04135-5

**Published:** 2025-10-25

**Authors:** Ignazio Maria Viola, Aditya Potnis, Soumarup Bhattacharyya, Evan J. Williams, Doug Halley, David Murphy

**Affiliations:** 1 School of Engineering, Institute for Energy Systems, University of Edinburgh, Edinburgh, EH9 3FB UK; 2 Department of Mechanical Engineering, University of South Florida, Tampa, FL 33620 USA

## Abstract

Understanding the gust response of free-falling bodies such as plant seeds and debris is critical in predicting their dispersal. Furthermore, gusts can significantly affect the performance and survivability of low-inertia aerial vehicles. However, current methodologies for studying common gusts, particularly transverse gusts, which are characterised by the sudden appearance of a flow velocity component orthogonal to the flyer’s velocity, are not applicable to untethered or free-falling bodies. This article introduces a novel approach that addresses this limitation through an accelerating reference frame generating a fictitious force that temporarily scales and redirects the gravitational force. This approach is demonstrated through a first-of-its-kind vertical wind tunnel that accelerates horizontally in the direction normal to the flow with the same acceleration as the gust. A preliminary characterisation of the facility is presented. The tunnel acceleration generates the same pressure gradient as irrotational, uniform transverse gusts, without introducing the shear layer typical of Küssner’s gusts. The gust response of a free-falling dandelion diaspore to a discrete transverse gust (Wagner type) is demonstrated, but the proposed approach is suitable for arbitrary time series of transverse gusts, including Theodorsen-type periodic gusts. For the first time, this novel approach will allow investigating the dynamic response of untethered bodies to transverse gusts, including micro- and nanodrones, unpowered microrobots, plant seeds, debris and more.

**Supplementary Information:**

The online version contains supplementary material available at 10.1007/s00348-025-04135-5.

## Introduction

Small flyers, including drones (Watkins et al. 2006; Chen et al. 2019) and natural flyers like insects (Vance et al. 2013; Gu et al. 2020; Zhang et al. 2023), seeds and spores (Nathan et al. 2002) experience disturbance to the incident flow due to turbulence, wind shear, convection, terrain and topography, including wakes of structures such as buildings and trees. Abrupt changes in the incidence flow, including in speed and/or in direction, are referred to as gusts.

The impact of gusts depends significantly on the inertia and relative velocity of the flyer with respect to the flow disturbance. For large aircraft, gust velocities are typically of much lower magnitude relative to their flight speed. Nonetheless, severe gusts can induce high transient loading on the airframe and wings, raising concerns about safety and operability (Wu et al. 2019). Consequently, much of the research on gust effects in aviation focuses on predicting these forces and developing strategies to mitigate them, ensuring sustained, smooth flight without the risk of stall (Frederick et al. 2010; Al-Battal et al. 2019; Andreu Angulo and Babinsky 2021; Sedky et al. 2020; Li and Qin 2022; Liu et al. 2024). Wind tunnel tests are typically conducted by varying the flow speed and direction over a fixed model or by prescribing the model’s kinematics to replicate the same relative velocity between the vehicle and the flow disturbance.

For smaller flyers, where gust velocities often match or exceed their characteristic flight speeds, gusts can dramatically alter the flight dynamics, including the orientation, position and trajectory of the flyer (Ravi et al. 2015; Pines and Bohorquez 2006; Ol et al. 2008). For microaerial vehicles (MAVs), for example, studying gust encounters is critical for designing effective control systems to ensure stability, efficiency and manoeuvrability (Floreano and Wood 2015; Di Luca et al. 2020; Kambushev et al. 2019; Mohamed et al. 2023).

Gusts play a significant role also in the dispersal and evolutionary behaviour of plant diaspores, pollen and spores. These passive natural flyers are capable of travelling vast distances and depend on wind gusts for dispersal. The study of the gust response of these passive flyers is necessary for gaining insights into plant adaptations and for tackling conservation, ecological and agricultural challenges.

Natural flyers are increasingly inspiring the design of flying microrobots. Some examples include those inspired by the diaspore of the dandelion (*Taraxacum officinale*), the maple (*Acer palmatum*), the Javan cucumber (*Alsomitra macrocarpa*) and the milkweed (*Asclepias*) (Nathan et al. 2002; Viola and Nakayama 2022; Galler and Rival 2021; Cummins et al. 2018). Understanding gust response allows increasing the endurance and range of plant-seed-inspired flying microrobots, which have a wide range of applications including environmental monitoring, responding to natural disasters, nuclear powerplant decommissioning, short-term weather forecasting (nowcasting), agrisecurity, water resource management, surveillance and recognition (Kim et al. 2021; Iyer et al. 2022; Chen et al. 2023; Wang et al. 2023; Yang et al. 2024).

Gusts can be broadly classified as either continuous or discrete. Continuous gusts include, for example, the spectrum of flow disturbances typically present in atmospheric turbulence. These are often modelled as stochastic processes, described using spectral distributions such as the von Kármán model or Dryden model (Beal 1993; Solari 1987)). Discrete gusts, on the other hand, are isolated single events. Notably, continuous gusts can be approximated as a series of discrete gusts (Zbrożek 1965). Studies on discrete gusts have been extensively reviewed in Jones et al. (2022). In the present work, we primarily focus on discrete gusts.

Canonical discrete gusts that have been widely investigated are streamwise, vortex and transverse gusts. Streamwise gusts are flow velocity disturbances along the freestream or flight direction (Ma et al. 2021; He and Williams 2020; Takeuchi and Maeda 2013). Vortex gusts are localised disturbances caused by passing vortices, often arising from wakes or interactions with turbulent eddies (Peng and Gregory 2015; Rockwood and Medina 2020; Biler et al. 2019b; Hufstedler and McKeon 2019; Harding et al. 2014). This work focuses on transverse gusts, which are lateral disturbances in the flow velocity perpendicular to the direction of motion. These disturbances arise from, for example, turbulence and particular weather conditions (Leishman 1996; Corkery et al. 2018; Volpe et al. 2013; Humphreys 1995). A transverse gust is, for example, a downward gust, which can potentially suddenly decrease a MAV’s altitude. For a free-falling body, a transverse gust is a horizontal wind fluctuation.

**Fig. 1:**
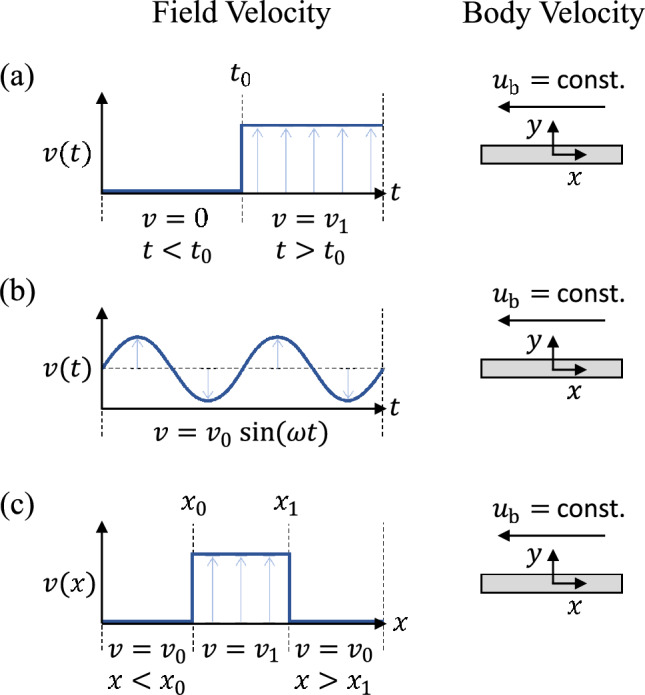
Illustration of flat plate moving at a constant horizontal speed $u_\text {b}$ experiencing different types of transverse gusts: (**a**) Wagner’s gust, (**b**) Theodorsen’s gust and (**c**) Küssner’s gust

Linear models of transverse Gusts were first developed by Wagner (1924). His work allows the computation of the transient lift experienced by a thin aerofoil with a small camber at a small angle of attack, for which quasi-steady thin aerofoil theory assumptions apply, to a step change in the transverse flow velocity. For example, this model is applicable to a flat plate moving at a constant horizontal speed $u_0$ in a flow field where the vertical flow velocity instantaneously varies from $v=0$ to $v=v_1$ (Fig. 1a). Glauert (1930) and Theodorsen (1934), and successively Atassi (1984), considered a periodic variation of the transverse velocity and found the solution in the frequency domain (Fig. 1b). While these models consider a uniform change of the transverse flow velocity along the aerofoil, Küssner (1936), and then von Karman and Sears (1938), considered a sharp-edged transverse gust, where the aerofoil gradually enters into the gust and the transverse velocity is first experienced by the foil’s leading edge and ultimately by the trailing edge (Fig. 1c). Despite the remarkable applicability of these models to a wide range of conditions, they are inadequate in cases of strong gusts because they do not account for flow separation and the effect of the body-generated vorticity on the gust (Perrotta and Jones 2017; Grubb et al. 2020; Andreu-Angulo et al. 2020; Biler et al. 2019; Ōtomo et al. 2021). Gust strength is typically quantified using the gust ratio, $G_\text {R}$, which is the ratio of the maximum velocity of the gust with respect to the velocity of the flyer. Therefore, the investigation of large-amplitude gusts, which have large $G_\text {R}$ that induce high angles of attack, flow separation, vortex-dominated flows or coupled interactions between the gust and flyer, requires experimental studies to uncover the underlying mechanisms.

Experimental studies of transverse gusts are typically carried out in wind or water tunnels to measure the forces and the flow field around constrained bodies, whose kinematics is prescribed. An overview of the facilities utilised for experimental studies of transverse gusts is presented in Table 1. These set-ups are often customised for investigating specific conditions, with a large number focusing on high *Re* flows in wind tunnels and employing various gust generators. A common approach is plunging a model into a uniform constant flow stream (Arredondo-Galeana et al. 2021). This experimental approach allows replicating both individual gusts and periodic gusts corresponding to Wagner’s (Wagner 1924) and Theodorsen’s (Theodorsen 1934) pure plunging models, respectively. Alternatively, instead of plunging a model in a uniform constant stream, it can be towed and plunged in a quiescent flow (Perrotta and Jones 2018; He and Williams 2020). Notably, in both these cases, the only vorticity in the flow is that generated by the model, and the model experiences a change in the transverse flow velocity that is uniform. The limitation of these experimental methodologies is that they require tethering the model, whose plunging motion is prescribed a priori. While the forces and moments experienced by the model in response to the gust can be measured, it is not possible to study how the body would move in response to these loads, and thus the complex fluid–structure interaction of the dynamic response of the model to the gust.

This limitation can be mitigated by controlling the kinematics of the body in real time through a feedback-controlled robotic system, which responds to the loads that the body experiences. This is known as cyber-physical testing (Mackowski and Williamson 2011; Williamson 2019). This approach is excellent for programming and automating the tests procedure, and thus exploring wide parameter spaces. It has been applied successfully to cylinders (Mackowski and Williamson 2013), gliders (Fagley et al. 2016) and aerofoils (Mackowski and Williamson 2017). However, applying such systems to ultralight flyers, such as plant diaspores and microdrones, is technically challenging due to the small aerodynamic forces that should be measured and that are needed to compute and prescribe the resulting kinematics. Furthermore, for three-dimensional fully immersed bodies, the robotic arm holding the body would interfere with the flow field. This issue does not occur when testing extruded models with a nominally infinite span, as these can be held from an end extending outside of the test section. For example, extruded models piercing the water can be held from a dry arm outside of the water.

Other methodologies to model transverse gusts rely on dynamically bending the streamlines. This can be achieved, for example, by bending the side walls of a water or wind tunnel or by including different obstructions on opposite sidewalls (Holmes 1973; Wooding and Gursul 2003; He et al. 2021; Fernandez et al. 2021, 2022). If the vorticity is confined in the boundary layers near the sidewalls, the gust is vorticity-free. While these methodologies are applicable to untethered models, the gust is not exactly uniform as the streamlines are inherently curved.

In conclusion, there is no methodology that allows testing untethered models experiencing a uniform, irrotational transverse gust. Yet, studying these gusts is essential because they allow for isolating the effect of flow acceleration from that of shear in a complex gust that includes both effects, such as in turbulence. While intense accelerations often occur in vortex cores in which velocity gradients are high (Biferale et al. 2005), significant acceleration may also be due to sudden velocity changes in the relatively uniform flow regions outside intense shear layers (Ishihara et al. 2013).

Notably, these gusts are different from sharp-edged transverse gusts, such as those modelled by Küssner (1936). The latter can be studied, for example, by towing a model in a tank of quiescent flow and then through a tank section where a transverse current is generated via the blowing and suction of flow (Perrotta and Jones 2017; Andreu-Angulo et al. 2020; Biler et al. 2019). In this case, the model is towed through two shear layers bounding the transverse current. Hence, both in the theoretical model (Küssner 1936) and in the physical realisation, the vorticity is part of the background flow, and the gust is non-uniformly experienced by the model as it crosses these shear layers.

This paper presents a novel approach that allows for the investigation of the effects of uniform, irrotational transverse gusts on untethered bodies. The underlying principle is the acceleration of the volume of fluid around the floating body in the direction orthogonal to its velocity. The flow acceleration is associated with a spatially uniform pressure gradient without shear. Unlike previous studies, this approach is implemented by accelerating the entire wind tunnel rather than a part of the flow field or the model. The approach is inherently scale-agnostic, applicable from particles in microfluidic systems to drones, provided the working fluid behaves as a continuous incompressible fluid. It allows precise control over the gust’s shape and intensity, and allows for the study of high gust ratios and large-amplitude gust responses. Furthermore, it allows the development of a relatively small wind tunnel compared to the size of the model because it limits the displacement of the model within the test section.

The rest of the paper is organised as follows. The novel approach is presented in Sect. 2. Here, we present the governing equations of a free-falling body in quiescent flow (Sect. 2.1) and then those of a free-falling body experiencing a uniform, irrotational transverse gust (Sect. 2.2). The comparison between these two sets of equations allows gaining insights on the effect of a transverse gust and how this can be modelled numerically or experimentally (Sect. 2.3). In Sect. 3, we present a practical implementation of the proposed approach with a horizontally accelerating wind tunnel, including the objectives (Sect. 3.1) and specifications (Sect. 3.2) of the facility, along with its characterisation while stationary (Sect. 3.3) and accelerating (Sect. 3.4). Finally, we demonstrate the proposed approach with an experimental test of a dandelion diaspore in Sect. 4. The key points of the methodology are summarised in Sect. 5. In Appendix A, we derive the non-dimensional set of equations used in Sect. 2.

**Table 1:** Overview of facilities utilised for experimental studies of transverse gusts

Organisation	Facility^2^	Gust type	Method^3^	Gust vorticity^1^
Army Research Lab, USA. Smith et al. (2018), Stutz et al. (2022)	WT	Sharp-edged	3	–
Institut Supérieur de l’Aéronautique et de l’Espace (ISAE), France. Volpe et al. (2013)	WT	Sharp-edged	3	–
Duke University, USA. Tang & Dowell (1995)	WT	Smooth	1	–
Michigan State University, USA. Olson et al. (2021)	WT	Smooth	2	–
Middle East Technical University, Turkey. Yigili et al. (2022)	WT	Smooth	5	–
Office National d’Études et de Recherches Aérospatiale (ONERA), France.				
Lepage et al. (2015), Brion et al. (2015)	WT	Smooth	1	–
Delft University of Technology, Netherlands. Lancelot et al. (2017)	WT	Smooth	5	–
Swansea University, UK. Balatti et al. (2022, 2023)	WT	Smooth	2	–
Cranfield University, UK. Saddington et al. (2015)	WT	Smooth	5	–
Politecnico di Milano, Italy Ricci et al. (2017), Fonte et al. (2016)	WT	Smooth	5	–
Technical University of Darmstadt, Germany. Rival et al. (2009), Wei et al. (2019)	WT	Smooth	7	–
University of Oldenburg, Germany. Knebel et al. (2011), Wei et al. (2019), Singh et al. (2024)	WT	Smooth	5	–
German Aerospace Center(DLR), Germany.				
Mulleners and Raffel (2012), Neumann and Mai (2013), Deparday (2019)	WT	Smooth	7	–
Beijing University of Aeronautics and Astronautics, China. Wang and Feng (2022), Wang et al. (2024)	WT	Smooth	5	–
Nanjing University of Aeronautics and Astronautics, China. Wu et al. (2019)	WT	Smooth	5	–
University of Bath, UK. Young and Smyth (2021), Fernandez et al. (2021), Bricker et al. (2025)	WT	Smooth	1	Irrotational
Cambridge University, UK. Holmes (1973)	WT	Smooth	8	Irrotational
Illinois Institute of Technology, USA. He et al. (2021, 2022)	WT	Smooth	6	Irrotational
University of Maryland, USA.				
Perrotta and Jones (2017); Sedky et al. (2020, 2022), Biler et al. (2021),	TT	Smooth	3,4	–
Cambridge University, UK.				
Corkery et al. (2018); Andreu-Angulo et al.(2020); Gehlert & Babinsky (2021); Andreu-Angulo & Babinsky (2023)	TT	Sharp-edged	3	–
Queen’s University, Canada. El Makdah et al. (2019, 2019), Burelle et al. (2020)	TT	Smooth	2	–

^1^In the fifth column, gust vorticity refers to vorticity accompanying gust generation, – denotes production of vorticity, Irrotational denotes no production of vorticity

^2^In the second column, WT represents wind tunnel, and TT represents a towing tank with water as ambient fluid

^3^For gust methods in the fourth column, 1 denotes tunnel flow blocked by shutters/vanes, 2 denotes gust-producing upstream disturbance, 3 denotes jets normal to main relative freestream, 4 denotes the moving model, 5 denotes gust generated by oscillating aerofoil/hydrofoils/vanes, 6 denotes the addition of suction duct on the top of the test section, 7 denotes pitching/plunging of model, 8 denotes oscillating tunnel walls.

## Theoretical analysis of the proposed approach

Consider a solid body moving with velocity $\varvec{ u}_\text {b}$ in an incompressible fluid (Fig. 1a). An irrotational, uniform, transverse gust is the acceleration of the background flow, i.e. of the far-field fluid, in the direction orthogonal to $\varvec{ u}_\text {b}$. For example, consider a body settling vertically due to gravity. In Sect. 2.3, we will show that if the density of the body is the same as that of the fluid, the body accelerates horizontally with the same acceleration as the far-field fluid. Instead, if the density of the solid is lower or higher than that of the fluid, then the solid accelerates faster or slower than the far-field fluid, respectively, and a relative transverse velocity is established between the body and the fluid. In a frame of reference fixed with the far-field fluid, the body is displaced horizontally by a body force that is equal to the difference between its own inertia and the inertia of the volume of fluid that it has displaced.

The proposed approach aims to enable the study of the dynamic response of an untethered body to the horizontal acceleration established because of the inertia difference between the fluid and the body. This is achieved by accelerating a volume of fluid surrounding an untethered solid body. For a free-falling body, the body may be made to hover at a constant height in a constant vertical airstream enclosed by solid walls. Then the walls, and thus the entire enclosed airstream, are accelerated horizontally at the gust acceleration.

### Governing equations of a free-falling body in quiescent flow

We formally derive the governing equations of a free-falling body in a non-inertial frame of reference that moves with the tunnel. The governing equations also provide a numerical framework to model the experiments in the translating wind tunnel with computational fluid dynamics.

First, consider the governing equation in an arbitrary inertial frame P$(\hat{x},\hat{y},\hat{z})$, where the hat above the symbols is used for dimensional quantities. To non-dimensionalise the governing equations, we use the fluid density $\hat{\rho }_\text {f}$, a reference length ${\hat{l}}$ and the gravitational velocity $\hat{u}_g\equiv ({\hat{m}} {\hat{g}}^*/ \hat{\rho }_\text {f} {\hat{l}}^2)^{1/2}$, where ${\hat{m}}$ is the mass of the body and ${\hat{g}}^* \equiv (1-\hat{\rho _\text {f}}/\hat{\rho _\text {b}}) {\hat{g}}$ is the reduced gravitational acceleration, which is the magnitude of the gravitational acceleration net of the specific buoyancy force, with $\hat{\rho _\text {f}}$ and $\hat{\rho _\text {b}}$ being the fluid and body mean density, respectively. Notably, this non-dimensionalisation requires ${\hat{u}}_g \ne 0$, and, thus, $\hat{\rho _\text {f}}/\hat{\rho _\text {b}} \ne 1$. The governing equations are the continuity equation (equation 2.1), the Navier–Stokes equation (equation 2.2) and the two Euler’s laws of motion for a rigid body stating the conservation of linear (equation 2.2) and angular (equation 2.4) momentum, respectively. Their non-dimensional forms are (see derivation in Appendix A)

2.1 $$\begin{aligned} & \nabla \cdot \varvec{u} = 0, \end{aligned}$$

2.2 $$\begin{aligned} & \quad \frac{\partial \varvec{u}}{\partial {t}} + (\varvec{u}\cdot \nabla )\varvec{u} = -\nabla {p} + \frac{1}{ Ga}\nabla ^{2}\varvec{u}, \end{aligned}$$

2.3 $$\begin{aligned} & \quad m \varvec{\dot{u}}_\text {b} =\varvec{F}+\varvec{\gamma }, \end{aligned}$$

2.4 $$\begin{aligned} & \quad \mathbb {I}_\text {o}\varvec{\dot{\omega }}_\text {b} +\varvec{\omega }_\text {b} \times \mathbb {I}_\text {o} \varvec{\omega }_\text {b} =\varvec{T}_\text {o} +e\varvec{r} \times \varvec{\gamma }{{/(\rho -1)}}, \end{aligned}$$

where $\varvec{u}$ is the velocity vector as observed by an arbitrary inertial frame P(*x*, *y*, *z*) such as, for example, Earth-fixed; *t* is time; $p\equiv p_\text {ph}-g\eta$, is the physical pressure ($p_\text {ph}$) net of the hydrostatic pressure ($g\eta$), with $\eta$ being a vertical coordinate in the direction of $\varvec{g}$ with origin where the hydrostatic pressure is zero; $Ga \equiv \hat{u}_g {\hat{l}} /\hat{\nu }$ is the Galilei number, with $\hat{\nu }$ being the fluid kinematic viscosity; *m* is the mass of the body; $\varvec{u}_\text {b}$ is the velocity of the centre of gravity of the rigid body with respect to P; $\mathbb {I}_\text {o}$ is the inertia tensor with respect to a non-inertial body-fixed frame centred at the centre of gravity O$(\tilde{x},\tilde{y},\tilde{z})$; $\varvec{F}$ is the fluid force exerted on the body; $\varvec{\omega }_\text {b}$ is the angular velocity of the solid body with respect to P; $\varvec{T}_\text {o}$ is the fluid torque about O; $\varvec{\gamma }$ is a unit vector in the direction of $\varvec{g}$; *e* is the Euclidean distance between the centres of gravity and buoyancy; and $\varvec{r}$ is a unit vector from the centre of gravity to the centre of buoyancy, and $\rho \equiv \hat{\rho _\text {f}}/\hat{\rho _\text {b}}$ is the density ratio.

The reader may be more familiar with the Reynolds number, *Re* instead of *Ga* in equation 2.2 (Batchelor 2000), but here we use *Ga* as the momentum equation is non-dimensionalised using the velocity scale $\hat{u}_g$, while we define *Re* based on the terminal velocity $\hat{u}_\text {t}$. The presence of *Ga*, which is the ratio between the gravity and the viscous forces, is a reminder of how the gravitational force influences the fluid equations for a free-falling body. When physical or numerical experiments are performed, $\hat{u}_\text {t}$ and thus *Re* are not known a priori, while the Galilei number is known, making it a more convenient control parameter (Brandt and Coletti 2022).

Equations 2.1 and 2.2 require initial and boundary conditions for the velocity and the pressure. For example, within the volume of the wind tunnel test section, suitable boundary conditions are Dirichlet boundary conditions: $\varvec{u}=0$ on the tunnel’s side walls and the surface of the floating body; $\varvec{u}=\varvec{u}_\text{inlet}$ on an upstream section of the tunnel, where the flow velocity is assumed to be known and is $\varvec{u}_\text{inlet}$; and $p=p_\text {out}$ at a downstream section of the wind tunnel, where the pressure is known and is $p_\text {out}$. The pressure *p* computed as a solution of equations 2.1 and 2.2 is the physical pressure, net of the hydrostatic pressure due to gravity. The physical pressure is not necessary for computing the aerodynamic force ($\varvec{F}$) and torque ($\varvec{T}_\text {o}$) on the body and, thus, for solving equations 2.3 and 2.4. However, it could be computed a posteriori as

2.5 $$\begin{aligned} p_\text {ph} =p+g\eta =p-g(x-x_\text {out}), \end{aligned}$$

where *x* is a streamwise, upward vertical coordinate and $x_\text {out}$ is the outlet section coordinate.

Equations 2.1 to 2.4 highlight the parameters governing the dynamics of a free-falling rigid body in an incompressible flow and a Newtonian fluid. The aerodynamics equations 2.1 to 2.2 are entirely characterised by *Ga*, and the kinematic equations 2.3 to 2.4 by $\rho , m$ and $\mathbb {I}_\text {o}$. Notably, for a rigid body with a uniform mass distribution, *m* and $\mathbb {I}_\text {o}$ are the sole function of $\rho$, i.e. $m=\rho \chi$ and each *i*, *j*th component of $\mathbb {I}_\text {o}$ is $[\mathbb {I}_\text {o}]_{i,j}=\rho \chi _\text {i,j}$, where $\chi$ and each $\chi _\text {i,j}$ depend only on the geometry. For example, for a disc, $\chi =\pi {\hat{f}} / {\hat{l}}$, where ${\hat{f}}$ is the plate thickness. Therefore, the free fall of a body with uniform mass distribution depends only on *Ga* and $\rho$.

### Governing equations of a free-falling body in a transverse gust

In this section, the governing equations for a free-falling body experiencing a transverse gust are derived. These are presented in a non-inertial frame that translates with the velocity of the gust. To derive these equations, first consider a generic non-inertial frame of reference with origin G($x',y',z'$) that translates with a linear velocity $\varvec{u}_\text {G}$ and rotates with an angular velocity $\varvec{\omega }_\text {G}$ with respect to a generic inertial frame P(*x*, *y*, *z*). Let the position, velocity and acceleration with respect to the non-inertial frame be indicated with a prime symbol ($'$). The relationships between the velocity and acceleration for the two frames of reference are, respectively:

2.6 $$\begin{aligned} & \varvec{u} =\varvec{u}_\text {G}+\varvec{u'}+\varvec{\omega }_\text {G} \times \varvec{x'}, \end{aligned}$$

2.7 $$\begin{aligned} & \quad \varvec{\dot{u}} =\varvec{\dot{u}}_\text {G}+\varvec{\dot{u}'}+2\varvec{\omega }_\text {G} \times \varvec{u'} +\varvec{\dot{\omega }}_\text {G} \times \varvec{x'}+\varvec{\omega }_\text {G}\times (\varvec{\omega }_\text {G}\times \varvec{x'}). \end{aligned}$$

The last three terms of equation 2.7 are, from left to right, the Coriolis acceleration, the Euler acceleration and the centripetal acceleration.

Now let G be the non-inertial frame fixed with the wind tunnel, which translates horizontally at the gust velocity $\varvec{u}_G$ and gust acceleration $\varvec{\dot{u}}_G$. Because the frame G has null angular velocity ($\varvec{\omega }_\text {G}=0$), the Coriolis acceleration, the Euler acceleration and the centripetal acceleration are null. Therefore, equations 2.6 and 2.7 become $\varvec{u}=\varvec{u}_\text {G}+\varvec{u'}$ and $\varvec{\dot{u}}=\varvec{\dot{u}}_\text {G}+\varvec{\dot{u}'}$. Substituting these equalities into equation 2.2, one finds

2.8 $$\begin{aligned} \frac{\partial \varvec{u'}}{\partial {t}} + (\varvec{u'}\cdot \nabla )\varvec{u'} = -\nabla {p} + \frac{1}{ Ga}\nabla ^{2}\varvec{u'} -\varvec{\dot{u}}_\text {G}. \end{aligned}$$

Comparing equation 2.8 with equation 2.2, we infer that the effect of a gust is equivalent to a body force $-m \varvec{\dot{u}}_\text {G}$. This force varies with time but is uniform in space, resulting in a uniform pressure gradient parallel to $\varvec{\dot{u}}_\text {G}$. The net effect of a uniform body force and the uniform pressure gradient that it generates is only to change the fluid pressure, but it does not propel the fluid, similar to the hydrostatic pressure due to the gravitational acceleration (see discussion in Appendix A). In the same way as the gravitational acceleration does not appear explicitly in equation 2.2 when the pressure *p* is the pressure net of the hydrostatic pressure due to gravity, equation 2.8 can be written in the same form as equation 2.2 by removing $\varvec{\dot{u}}_\text {G}$ and using a pressure $p'$ net of the hydrostatic pressure due to the gravitational acceleration as well as the hydrostatic pressure due to the gust acceleration. The hydrostatic pressure due to the gust acceleration is $-\dot{u}_\text {G}\epsilon$, where $\epsilon$ is a coordinate in the direction of the body force, $-\dot{\varvec{u}}_\text {G}$, with origin where the hydrostatic pressure is zero.

Therefore, substituting $\varvec{u}=\varvec{u}_\text {G}+\varvec{u'}$ and $\varvec{\dot{u}}=\varvec{\dot{u}}_\text {G}+\varvec{\dot{u}'}$ in the aerodynamic non-dimensional governing equations 2.1 and 2.2, and substituting $p=p'-u_\text {G}\epsilon$ in equation 2.2, one finds

2.9 $$\begin{aligned} & \quad \nabla \cdot \varvec{u'} = 0, \end{aligned}$$

2.10 $$\begin{aligned} & \quad \frac{\partial \varvec{u'}}{\partial {t}} + (\varvec{u'}\cdot \nabla )\varvec{u'} = -\nabla {p'} + \frac{1}{ Ga}\nabla ^{2}\varvec{u'}. \end{aligned}$$

**Fig. 2:**
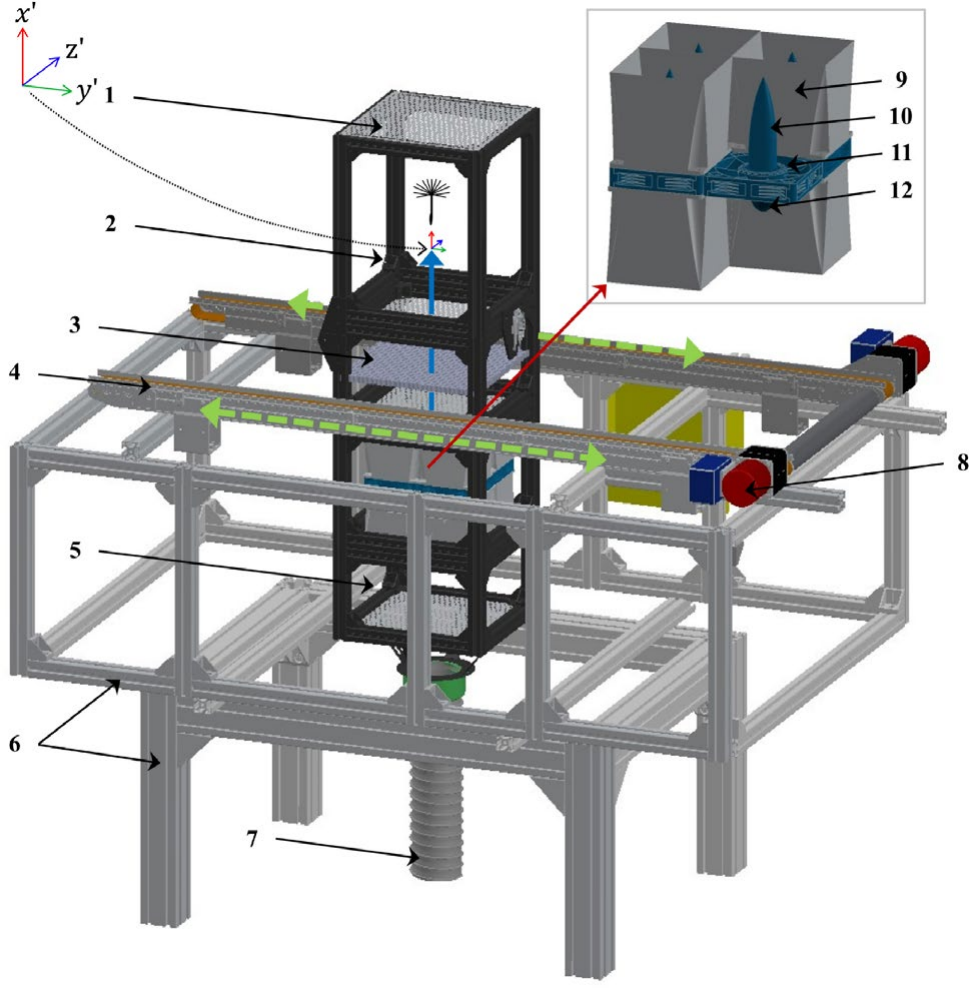
Illustration of the translating wind tunnel which traverses horizontally as shown by green arrows. Details: (1) wire cloth mesh, (2) test section showcasing dandelion and direction of airflow, (3) honeycomb structure, (4) motorised linear actuator, (5) settling chamber, (6) support frame, (7) seeding pipe, (8) stepper motors, (9) fan enclosure, (10) cone structure, (11) fan array, (12) dome structure.

Recalling that the equations are written for a frame that moves with the tunnel, suitable boundary conditions for the volume corresponding to the wind tunnel test section are: $\varvec{u'}= 0$ on the tunnel’s side walls and the surface of the floating body (if any); $\varvec{u'}=\varvec{u'}_\text {inlet}$ on the upstream, inlet section; and $p'=p'_\text {out}$ on the downstream, outlet section. This set of boundary conditions is the same as those for equations 2.1 and 2.2. However, the physical pressure is different because it now includes the horizontal hydrostatic pressure:

2.11 $$\begin{aligned} p_\text {ph} = p' + g\eta - \dot{u}_\text {G}\epsilon = p' -g(x'-x'_\text {out}) -u_\text {G}y' \end{aligned}$$

where $x'$ is the streamwise, vertical upward coordinate (Fig. 2), and $y'$ is a horizontal coordinate in the direction of $\varvec{u}_\text {G}$, centred in the tunnel section.

Because both aerodynamics equations 2.1 and 2.2 and their boundary conditions for a free-falling body in quiescent flow are the same as equations 2.9 and 2.10 for a free-falling body in the presence of a gust, we infer that the transverse gust does not change the flow field. This will be demonstrated experimentally in Sect. 3.4.

The aerodynamic force $\varvec{F}$, which can be computed from the solution of the aerodynamic equations 2.1 and 2.2, does not include the gravitational and buoyancy force, which are instead represented by $\varvec{\gamma }$ in the Euler’s equations 2.3 and 2.4. In Sect. 2.1, instead of including both the gravitational force and the buoyancy force, we considered only their net effect by defining the reduced gravitational acceleration, which is the gravitational acceleration net of its specific buoyancy force (see details in Appendix A ). We can take the same approach with the gust acceleration and define a gust acceleration net of its specific buoyancy force: $- \hat{\dot{\varvec{u}}}_\text {G}^* \equiv - (1-\hat{\rho }_\text {f}/\hat{\rho }_\text {b}) \hat{\dot{\varvec{u}}}_\text {G}$, whose non-dimensional form is $-\varvec{\dot{u}}_\text {G}^* \equiv (1/\rho -1) \varvec{\dot{u}}_\text {G}$. The specific buoyancy of the gust can be written either as $- \varvec{\dot{u}}_\text {G}/\rho$ or as $- \varvec{\dot{u}}^*_\text {G}/(\rho -1).$ Hence, the torque generated by the gust buoyancy force is, for instance, $-e \varvec{r} \times m\varvec{\dot{u}}^*_\text {G}/(\rho -1)$.

With this approach, Euler’s equations of motion become

2.12 $$\begin{aligned} & m \varvec{\dot{u}'}_\text {b} =\varvec{F}+\varvec{\gamma }-m\varvec{\dot{u}}^*_\text {G}, \end{aligned}$$

2.13 $$\begin{aligned} & \quad \mathbb {I}_\text {o}\varvec{\dot{\omega }}_\text {b} +\varvec{\omega }_\text {b} \times \mathbb {I}_\text {o} \varvec{\omega }_\text {b} =\varvec{T}_\text {o} +e\varvec{r} \times \varvec{\gamma }{{/(\rho -1)}} -e \varvec{r} \times m\varvec{\dot{u}}^*_\text {G}{{/(\rho -1)}} \end{aligned}$$

Euler’s equations of motion for a free-falling body in quiescent flow, equations 2.3 and 2.4, are different from equations 2.12 and 2.13 for a free-falling body in the presence of a gust. The difference is the additional body force $-m \varvec{\dot{u}}^*_\text {G}$ that changes the linear momentum in equation 2.12, and its torque in the case of a body whose centres of mass and buoyancy do not coincide in equation 2.13. Therefore, we conclude that in a non-inertial frame moving with velocity $\varvec{u}_\text {G}$, the effect of the gust is not to change the flow field, but that of a reduced gust force $-m\varvec{\dot{u}}^*_\text {G}$ that pushes the body in the direction opposite to the gust, and a torque $-e \varvec{r} \times m\varvec{\dot{u}}^*_\text {G}$ $/(\rho -1)$.

### Discussion

Overall, this analysis reveals that a transverse gust can be studied by applying a uniform linear acceleration to a volume of fluid, in the direction orthogonal to the initial velocity of the body. An untethered body with a density ratio $\rho \ne 1$ will be displaced with respect to the accelerating frame because of its inertia. In the limit of $\rho \rightarrow 1$, the effect of the gust vanishes because the reduced gust acceleration, which is $(1/\rho -1) \varvec{\dot{u}}_\text {G}$ (Sect. 2.2), vanishes when $\rho \rightarrow 0$. In fact, in these conditions, the gust acceleration is equal in sign and opposite in magnitude to the gust buoyancy, independently of the size and geometry of the body, and of the strength of the gust. Indeed, the same conclusion applies to a streamwise gust, but not to a vortex gust or a sharp-edged transverse gust such as those modelled by Küssner (1936).

The effect of the gust can be interpreted as a temporary modification of the gravitational acceleration, where the modified gravitational acceleration is the vectorial difference of the physical gravitational acceleration and the gust acceleration, $\varvec{g}-\varvec{\dot{u}}_\text {G}$. For example, if the gust acceleration is horizontal, the magnitude of the modified gravitational acceleration is $(g^2+\dot{u}^2_\text {G})^{1/2}$ and the direction changes by $\tan (\dot{u}_\text {G}/g)$ away from the gust direction. Notably, in atmospheric turbulence, $\varvec{\dot{u}}_\text {G}$ can be of the same order of magnitude, or even larger than $\varvec{g}$ (Shaw 2003). Therefore, the effect of these gusts can be significant. Notably, these accelerations are much greater than the Coriolis acceleration due to the Earth’s rotation, which is the other fictitious force experienced by the body.

If the gust occurs while the flow around the body is steady such as while it falls stably in quiescent flow, it is not the change in the flow field and, thus, the fluid force $\varvec{F}$ and torque $\varvec{T_\text {o}}$ (equations 2.12 and 2.13) that perturbs this initial state. In fact, the gust can modify the fluid equations (equations 2.9 and 2.10) only through the boundary conditions and thus the position of the body. Hypothetically, if one held the body in a fixed position with respect to the non-inertial frame during the gust, the fluid loads would remain constant indefinitely. Instead, it is the change in the perceived gravitational acceleration $\varvec{g}-\varvec{\dot{u}}_\text {G}$ that perturbs the untethered body from its stable fall. Once the body begins to move with respect to the surrounding flow, then the fluid forces $\varvec{F}$, torque $\varvec{T_\text {o}}$ also vary and contribute to governing the gust response of the body. Therefore, this approach allows for the study of the transient fluid–structure interaction initiated by the gust.

For the numerical implementation of the proposed approach, the set of non-inertial equations 2.9, 2.10, 2.12 and 2.13 can be solved with computational fluid dynamics by prescribing a time varying gravitational acceleration in the structural solver. For example, with a segregated approach, the flow field is first computed by solving equations 2.9 and 2.10 with a Navier–Stokes solver, without including gravity. The computed fluid loads, $\varvec{F}$ and $\varvec{T}_\text {o}$, are used as the input to solve equations 2.3 to 2.4 with a structural solver, where the gravitational acceleration is used as input to the solver is, at every time step, the vectorial difference between the physical gravitational acceleration and the gust acceleration. The structural solver returns the updated position of the rigid body, for which a new updated flow field is computed.

Alternatively, the proposed approach can be implemented experimentally with by accelerating a volume of fluid. In this paper, we consider a horizontally accelerating, vertically blowing wind tunnel. The tunnel accelerates horizontally with the gust acceleration $\varvec{\dot{u}}_\text {G}$, while an array of fans is used to generate an upward airstream matching the terminal velocity $u_\text {t}$ of the free-falling body. Therefore, in the absence of horizontal acceleration, the body remains at a constant height in the tunnel section. When the tunnel is accelerated horizontally, the body experiences a horizontal body force $-m \varvec{\dot{u}}^*_\text {G}$. The flow velocity within the tunnel, including around the body, measured with respect to a wind tunnel-fixed frame, is that seen by an observer who moves downstream at the mean terminal velocity of the body and that moves horizontally at the sum of the mean horizontal wind velocity and the horizontal gust velocity. A physical realisation of the proposed wind tunnel is presented in Sect. 3, and an experimental demonstration of the proposed approach is presented in Sect. 4.

Notably, the present approach could also be implemented in several other ways, including a drop tower, a rotating test section, etc., where a volume of fluid is uniformly accelerated. The gust acceleration would be represented by the fictitious acceleration experienced by the flow-immersed body as observed by a non-inertial frame fixed with the volume of fluid. For example, a free-falling body within a drop tower, which falls with acceleration $\varvec{g}$, would experience an uplifting gust acceleration $\varvec{\dot{u}}_\text {G}=\varvec{g}$.

## The dandidrone wind tunnel

### Wind tunnel objectives

In this section, we describe a particular application of the proposed methodology aiming at the study of the gust response of dandelion diaspores and dandelion-inspired drones, *dandidrones* hereafter, which have similar size, weight and terminal velocity as their natural counterpart.

The terminal velocity ($\hat{u}_\text {t}$) of the dandelion is about 0.5 m s$^{-1}$ and its filamentous pappus has a diameter $\hat{l}=\mathcal {O}(10)$ mm (Cummins et al. 2018). Hence, fans were chosen to ensure accurate control of the flow velocity between 0.1 m s$^{-1}$ and 1 m s$^{-1}$, and a square test section of 250 mm $\times$ 250 mm was selected to ensure a negligible blockage ratio. This velocity range allows testing free-falling bodies with a Reynolds number between about 60 and more than 6000. Therefore, it is suitable to study the transition from steady to unsteady wakes for both impervious and permeable bodies. In fact, flow instabilities arise in the wake of an impervious free-falling solid body when $Re=\mathcal {O}(100)$ (Ern et al. 2012), where $Re \equiv \hat{u}_\text {t} \hat{l} /\hat{\nu }$. For permeable bodies such as the dandelion pappus, the critical *Re* increases gradually with increasing permeability up to $Re=\mathcal {O}(1000)$, beyond which the wake is unconditionally stable (Ledda et al. 2019).

### Wind tunnel description

The schematic of the horizontally translating vertical wind tunnel is shown in Fig. 2. The wind tunnel is divided into four main sections from bottom to top: the settling chamber, the fan array, the flow conditioning section and the test section. The settling chamber facilitates air entry into the fan array and provides a volume for the mixing of the seeding used for flow visualisation and particle image velocimetry (PIV). The flow conditioning section, located downstream of the fan array, includes a 12.7-mm-thick honeycomb structure with 3 mm sided hexagonal cells made of aluminium alloy. The honeycomb caps the maximum amplitude of the crossflow velocity. Both sides of the settling chamber, as well as the exit of the test section, are covered by a stainless steel wire cloth mesh with 0.9 mm wire diameter and a 3.3 mm aperture to ensure laminar airflow into and out of the tunnel and to minimise the effects of ambient disturbances. The test section, located downstream and above the flow conditioning section, has a length of 0.25 m, a width of 0.25 m and a height of 0.4 m. The test section is hinged on one side to allow easy access for inserting test objects. Additionally, all the sections of the tunnel are separated by stainless steel woven wire meshes (0.559 mm wire diameter with 1.98 mm aperture), which provide additional flow conditioning required to maintain uniformity.

The flow of air in the tunnel is facilitated by an array of four (layout of $2\times 2$) fans, each with a diameter of approximately 0.12 m operating at a maximum of 1050 rotations per minute, controlled by Arduino Uno. The fan array is designed in-house and is shown in the inset of Fig. 2. To optimise performance, the cone structure above the fan and the dome structure below the fan ensure maximum pressure recovery, improve efficiency and reduce aerodynamic losses. For flow visualisation and laser diagnostics, seeding particles such as smoke or di-ethyl hexyl sebacat are introduced into the settling chamber using a seeding generator and necessary tubing.

The tunnel can be translated using a motorised linear actuator from HepcoMotion, housed within a conveyor roller frame. The movement is driven by two 6A stepper motors (Nema 34 Frame E Series), which are controlled via an Arduino Due from the workstation. By adjusting the input signals to the motors, the translation of the wind tunnel can be precisely modulated to simulate various transverse gust profiles and gust ratios. Safety is ensured by sensors and emergency switches located at the extreme ends of the system, preventing over-translation of the tunnel.

**Fig. 3:**
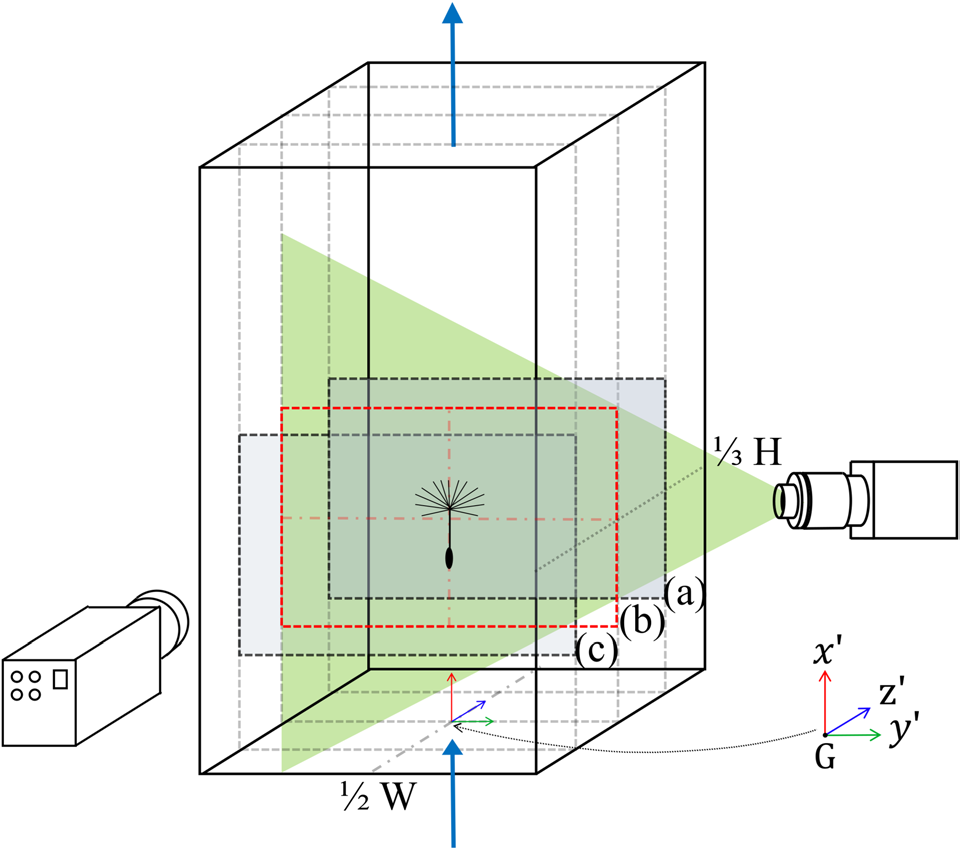
Schematic of the set-up for PIV illustrating the locations of three vertical cross sections of the wind tunnel test section at 1/4 (**a**), 1/2 (**b**) and 3/4 (**c**) of the tunnel depth, with (**c**) closer to the camera, corresponding to velocity fields shown in Fig. 4. The schematic also depicts the flow direction, the laser, sheet optics, the resulting laser sheet and the high-speed camera, which is positioned orthogonally to the sheet. The coordinate system is centred in the tunnel test section at 1/3 of the tunnel height (H) from the bottom screen and at the midpoint of its width (W)

**Fig. 4:**
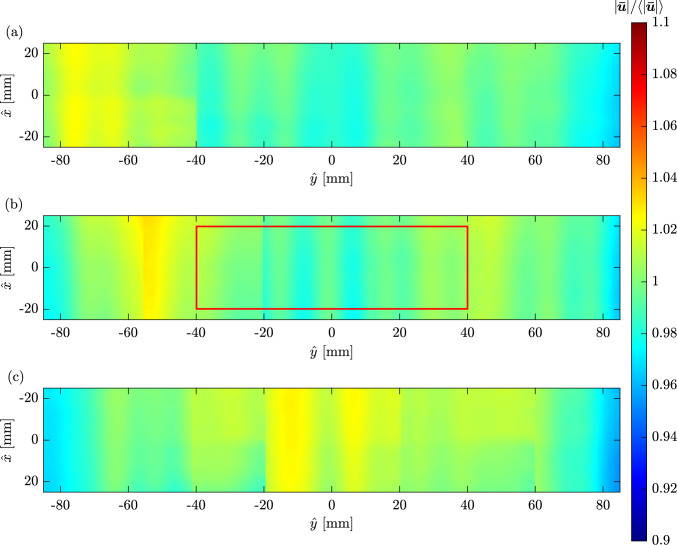
Magnitude of the time-averaged velocity divided by its mean across the three fields, on three vertical cross sections (illustrated in Fig. 3). The ${\hat{x}}$ axis is streamwise (vertical) and the ${\hat{y}}$ axis is crossflow (horizontal). The red area, measuring 40 mm high and 80 mm wide, is used to calculate the turbulent intensity

**Fig. 5:**
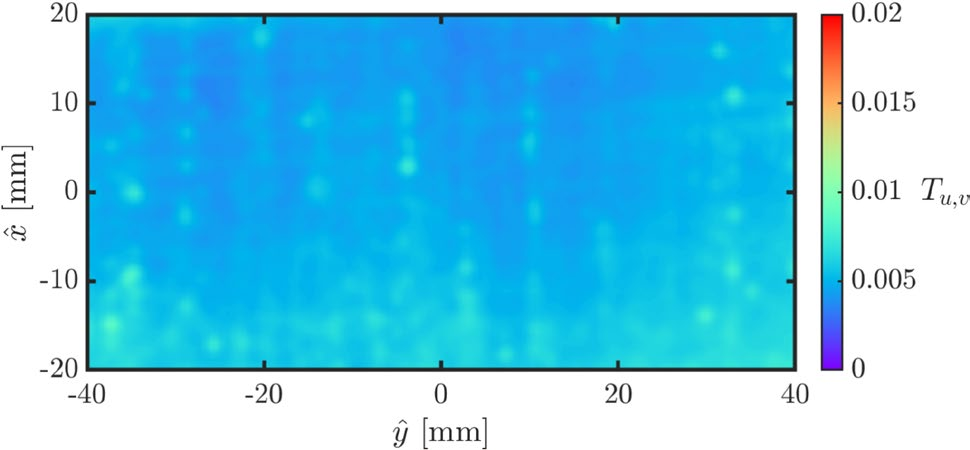
Turbulence intensity in the highlighted red area in Fig. 4.

### Wind tunnel characterisation

The velocity flow field in the wind tunnel was characterised using high-speed PIV, with acquisition details provided in the supplementary material. Figure 4 shows the contours of the magnitude of the time-averaged velocity, ${|\varvec{\bar{u}}|=|\overline{\varvec{u}(x,y)}|}$, divided by its mean value, $\left\langle {|\varvec{{\bar{u}}}|} \right\rangle$, across all three vertical cross sections (a–c in Fig. 3) for a stationary wind tunnel. The value of ${|\varvec{{\bar{u}}}|} / \left\langle {|\varvec{{\bar{u}}}|} \right\rangle$ varies by less than $\pm 2\%$ within the central volume of the test section spanned by the model during the experiments. Each time-averaged velocity field shown in Fig. 4 was constructed by combining three averaged velocity fields, obtained by shifting the camera position along *y*. The vertical lines visible in the vector fields coincide with the stitching boundaries and result from temporal differences between measurements used for averaging. Each stitched window spans approximately 6 cm to 8 cm in width, with overlap provided between adjacent windows to ensure continuity.

The turbulence intensity of the flow field for the static wind tunnel is quantified by the root mean square of velocity fluctuation,

3.1 $$\begin{aligned} T_{u,v}=\frac{1}{|\overline{\varvec{u}(x,y)}|}\sqrt{\frac{\overline{\left( u(x,y)-\overline{u(x,y)} \right) ^2} +\overline{\left( v(x,y)-\overline{v(x,y)} \right) ^2}}{2}}, \end{aligned}$$

where *u*(*x*, *y*) and *v*(*x*, *y*) are the instantaneous values of velocity components in the streamwise and streamnormal directions, respectively, and $\bar{u}$ and $\bar{v}$ denote their time-averaged values. The turbulence intensity was under $1\%$ at each of the three tested cross sections (a–c in Fig. 3). The measured $T_{u,v}$ in the central cross section (highlighted in red in Figs. 3 and 4) is shown in Fig. 5.

**Fig. 6:**
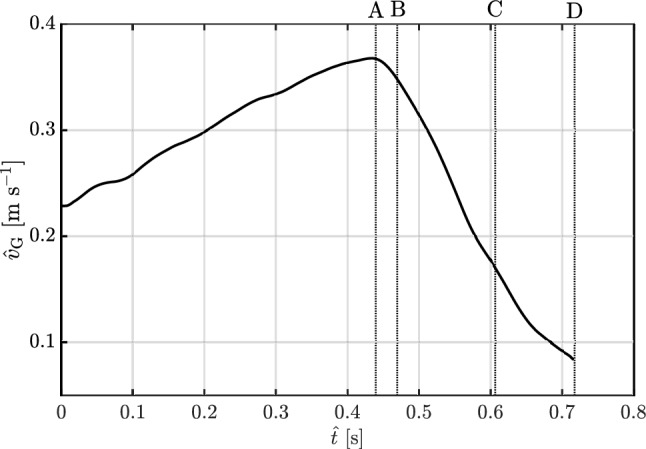
Temporal evolution of the horizontal speed of the translating wind tunnel. The time instants labelled **A**, **B**, **C** and **D** correspond to the moments at which the velocity field is presented in Fig. 7

**Fig. 7:**
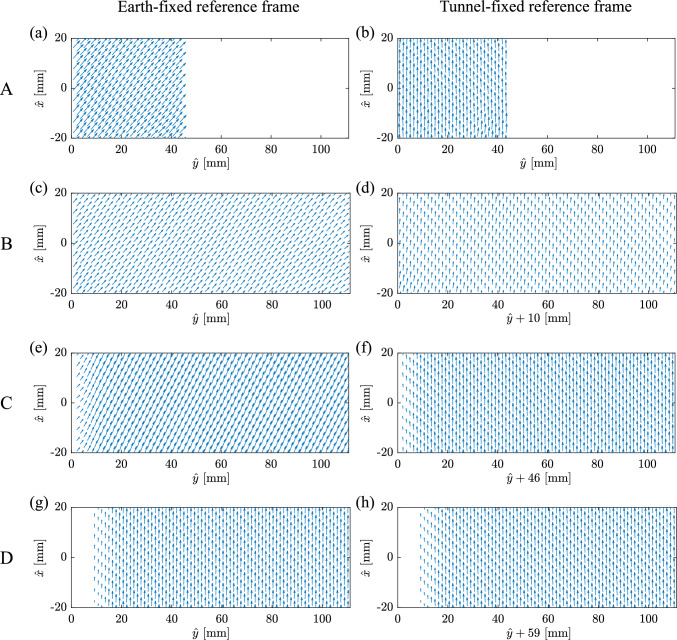
Instantaneous velocity vector fields at time **A** (**a**, **b**), **B** (**c**, **d**), **C** (**e**, **f**) and **D** (**g**, **h**) in the Earth-fixed frame of reference (**a**, **c**, **e**, **g**) and in the non-inertial frame fixed with the moving tunnel (**b**, **d**, **f**, **h**)

### Wind tunnel translation

To demonstrate the effect on the flow field of a linear acceleration of the wind tunnel, the latter was moved from left to right with a velocity profile shown in Fig. 6. This displacement, $y_\text {G}$, was determined by tracking a marker mounted on the rear of the tunnel using a high-speed camera operating at 1000 frames per second. The horizontal speed of the tunnel, $\hat{v}_\text {G}$, was measured by taking the time derivative of the de-noised displacement. The velocity vector field was obtained using high-speed PIV at 1000 frames per second, acquired in an Earth-fixed frame of reference.

The instantaneous velocity vectors corresponding to the time instants highlighted in Fig. 6A–D are presented in Fig. 7. The left column of Fig. 7a, c, e, g shows the vector fields in an Earth-fixed inertial frame P(*x*, *y*, *z*), while the right column (b,d,f,h) shows the fields in the non-inertial tunnel-fixed frame G($x',y',z'$). The velocity fields in the tunnel-fixed frame are derived from those measured in the Earth-fixed frame by subtracting the instantaneous velocity of the tunnel (i.e. not the smoothed value) obtained from the tracking data shown in Fig. 6, i.e. $\varvec{u}'=\varvec{u}-\varvec{u}_\text {G}$.

Figure 7 demonstrates that the vector field $\varvec{u}'$ observed in the non-inertial frame fixed to the tunnel remains unchanged under translation and acceleration of the tunnel, with no shear layers or velocity gradients. As described earlier in Sect. 2.2, the acceleration of the tunnel results in a uniform horizontal pressure gradient that does not change the flow field.

**Fig. 8:**
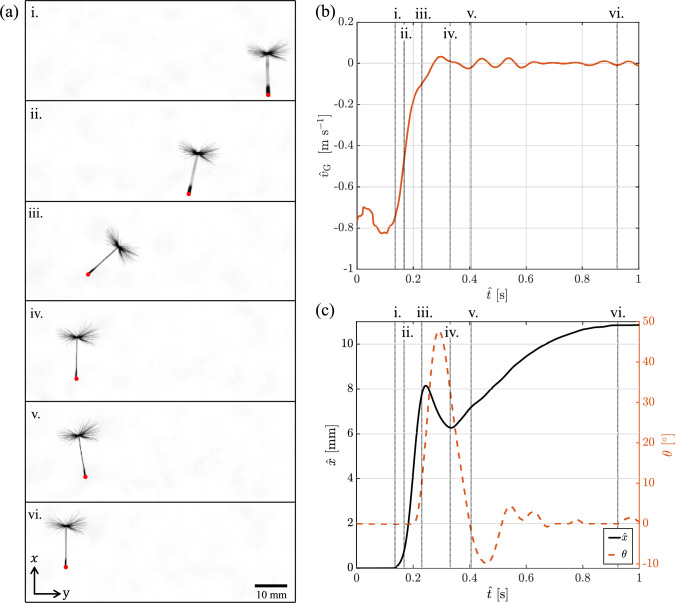
(**a**) Snapshots of the dandelion diaspore in flight as it experiences a transverse gust, imaged with a camera fixed in the Earth reference frame. (**b**) Gust profile: time evolution of the horizontal speed of the wind tunnel $\hat{v}_\text {G}$. (**c**) Time evolution of the change in the height of the dandelion diaspore $\hat{x}$ and its rotation ($\theta$) around the *z* axis

## Gust response of a dandelion diaspore

To illustrate the effect of a transverse gust on an untethered body, here we consider a free-falling dandelion diaspore experiencing a transverse gust (Fig. 8a). A diaspore that is passively transported by a constant and uniform horizontal wind moves horizontally at the wind velocity and downwards at the diaspore’s terminal velocity. The relative velocity between the diaspore and the wind is the terminal velocity. In the wind tunnel, the diaspore is kept suspended at a constant height by setting the vertical wind tunnel airflow to match the diaspore’s terminal velocity ($\hat{u}_\text {t}=0.38$ m s$^{-1}$ for the tested diaspore). Therefore, the initial stationary state of a diaspore floating at a constant height in the wind tunnel is that of a diaspore being carried by a constant and uniform horizontal wind with an arbitrary velocity.

Notably, this stationary state of the dandelion is achieved when $\varvec{\dot{u}}_\text {G}=\varvec{0}$, i.e. for a horizontally stationary wind tunnel as well as for any constant horizontal speed of the wind tunnel. In fact, we showed in Sect. 2.2 that the effect of the gust depends only on the acceleration and not on the velocity. Therefore, while the acceleration of the wind tunnel must match the acceleration of the gust ($\varvec{\dot{u}}_\text {G}$), the velocity of the wind tunnel can be arbitrarily set.

Consider now a sudden uniform variation in the horizontal wind speed and thus a uniform gust with acceleration $\varvec{\dot{u}}_\text {G}$. In Sect. 3.4, we demonstrated that the background flow field remains unchanged. Here, we show that the diaspore, which has a different density than air (and thus $\varvec{\dot{u}}^*_\text {G} \ne 0$), experiences a horizontal body force $-m\varvec{\dot{u}}^*_\text {G}$ and a torque $-e \varvec{r} \times m\varvec{\dot{u}}^*_\text {G}/{{(\rho -1)}}$ (Sect. 2.2). To replicate the wind gust, the wind tunnel is horizontally accelerated, ensuring that its acceleration matches the wind gust acceleration $\varvec{\dot{u}}_\text {G}$. As a gust example, we consider a sharp Wagner-type transverse gust (Fig. 1a), with a gust ratio of 2. The acceleration is achieved by translating horizontally the tunnel at $\hat{u}_\text {G}= -2 \hat{u}_\text {t}$ and then suddenly and rapidly decelerating it to a stop (Fig. 8b). Notably, accelerating the tunnel in one direction is equivalent to decelerating the tunnel in the opposite direction.

To capture the gust profile, the wind tunnel was tracked using high-speed imaging of a marker on the back panel. This imaging was performed at 750 frames per second at a resolution of 0.0805 mm per pixel. The images were analysed using the open-source imaging software Fiji (Schindelin et al. 2012). The track of the marker enabled the determination of the tunnel’s position. The instantaneous velocity of the wind tunnel was obtained by calculating the second-order time derivative of the marker’s position. This velocity was de-noised using a ten-point moving average filter. The second derivative of this smoothed velocity provided the instantaneous acceleration.

The dandelion diaspore was filmed using high-speed shadowgraphy with a high-speed camera and a backlight Fig. 8a. The diaspore was recorded at 750 frames per second with a resolution of 0.0805 mm per pixel using a high-speed camera. The camera was stationary in the Earth-fixed frame. As the tunnel and the dandelion entered and passed through the camera’s field of view, the camera’s snapshots captured the diaspore’s trajectory. These snapshots of the dandelion are shown in Fig. 8a with the corresponding times highlighted in Fig. 8b, c.

In the image analysis, the lowest tip of the dandelion diaspore (the tip of the achene or seed at the base, indicated by red dots in Fig. 8a) was tracked frame by frame to compile a time history of the change in its height ($\hat{y}_\text {b}$) during the gust event. Further, the centroid of the area of the shadowgraph, i.e. the profile of the diaspore, was tracked to obtain the displacement of the diaspore in the transverse direction. Its derivative provided its horizontal speed ($\hat{v}_b$). Additionally, to illustrate the change in orientation of the diaspore, the angle of the dandelion’s stem ($\theta$) relative to the vertical is obtained and tracked, with positive values indicating clockwise rotation. Details of image processing and tracking methodologies are provided in the supplementary materials.

As shown in Fig. 8a, at the initial state ($\hat{t} = 0$ s), the wind tunnel traverses with a mean velocity of approximately $-0.8$ m s$^{-1}$. At $\hat{t}=0.1$ s, the gust event is initiated, and the tunnel has an acceleration of approximately 11 m s$^{-2}$ before coming to a stop at 0.27 s. The gust response of the dandelion diaspore is shown in Fig. 8a, which shows different snapshots of the same field of view in a tunnel-fixed frame. Initially (time frame i), the dandelion is stationary on the right of the field of view. As the tunnel accelerates with a positive acceleration towards right, the diaspore is displaced towards the left. We demonstrated in Sect. 3.4 that the flow field is unchanged by the acceleration. Therefore, it is not the aerodynamic force and torque that make the dandelion move from its original position. Instead, the displacement towards left of the diaspore is initiated exclusively by the body force $-m\varvec{\dot{u}}^*_\text {G}$. As soon as the dandelion begins to move in the wind tunnel frame, the flow field around the dandelion changes in response to this. Consequently, the aerodynamic forces and torque also change because of the dandelion’s displacement, affecting the dandelion’s dynamics.

The centre of gravity of the dandelion diaspore is near the seed, on the lowest part of the diaspore, while the centre of buoyancy is near the centre of the filamentous pappus. Therefore, $\varvec{r}$ is from the centre of the pappus to the seed. When initially upright (i), before the tunnel accelerates, the torque due to the buoyancy, $e\varvec{r} \times \varvec{\gamma }/(\rho -1)$, is null because $\varvec{r}$ is parallel to $\varvec{\gamma }$. The torque due to the gust buoyancy, $-e \varvec{r} \times m\varvec{\dot{u}}^*_\text {G}/(\rho -1)$, is also null because $\varvec{\dot{u}}^*_\text {G}=0$. As the tunnel accelerates, $\varvec{\dot{u}}^*_\text {G}>0$, the torque due to the gust buoyancy results in a positive rotation around the *z* axis, which is into the plane, resulting in the clockwise rotation observed in Fig. 8a (ii-iii). As the acceleration ends (iv), the torque ends, and the diaspore turns upright because of the torque due to the buoyancy, $e\varvec{r} \times \varvec{\gamma }/(\rho -1)$. Eventually, the dandelion rights itself, reaching a new steady state at a higher axial location or increase in $\hat{y}$ i.e. indicating an altitude increase in response to the gust event as evident from Fig. 8c.

The transient translation and rotation of the dandelion diaspore with respect to the tunnel frame result in a relative velocity between the diaspore and the surrounding fluid, and thus in the generation of an aerodynamic force $\varvec{F}$ and a torque $\varvec{T}_\text {o}$. Notably, $\varvec{F}$ results in a transient upward force that produces a temporary reduction in the terminal velocity and thus an increase in height within the wind tunnel. A more detailed investigation into the wake dynamics, flow field and fluid mechanics underlying the dandelion’s response to gusts will be explored in future work and is beyond the scope of this article. The reported changes in flight trajectory, orientation and dynamics are the result of the transverse gust simulated by the horizontal translation of the wind tunnel, demonstrating the effectiveness of the system for studying gust–aerodynamic interactions.

## Conclusions

We presented a new approach to study the dynamic response of untethered bodies to transverse gusts. This is achieved by using the fictitious acceleration of a non-inertial frame to rescale and change the direction of the gravitational acceleration. The novelty of this approach lies in its ability to create uniform, irrotational gusts, and in enabling the study of the gust response of an untethered body, including its trajectory, orientation and the resulting fluid–structure interaction. Understanding the flyer’s response to this type of transverse gusts is crucial for control system design of small drones and microrobots, and to predict the dispersal of passive flyers such as plant diaspores and microrobots.

A novel facility based on this approach was built, characterised, and a dandelion diaspore was tested. Preliminary results show that a free-falling flyer, such as a dandelion diaspore, can experience a gain in altitude in response to a single transverse gust with a step change in velocity. This discovery opens up opportunities to study the flight of such flyers, which typically encounter multiple transverse gusts during flight, with the possibility of a sustained passive flight.

A limitation of the present approach is that it only allows testing gusts with zero shear. However, real gusts involve some flow shear. For example, a dandelion experiencing acceleration due to turbulent eddies will be subjected to both accelerating and sheared flow. While the advantage of this approach is that it allows for decoupling the effect of the acceleration from that of the shear, only zero shear flow can be easily tested.

In addition, the need to accelerate the entire volume of fluid around the untethered body may require significant power. For example, in the demonstrated horizontally accelerating wind tunnel, the power is delivered by a motorised linear actuator. The power requirement increases with the weight of the wind tunnel and the friction in the linear actuators, effectively limiting the maximum acceleration that can be generated. However, in addition to the discrete step change in velocity presented in this study, a wide range of gust functions can potentially be explored with the presented approach. For example, the motor could be programmed to execute smoother functions, or specific time histories with specific energy spectra to mimic turbulent gusts. Finally, this approach could potentially be used to combine transverse and streamwise gusts through rapid modulation of the fan speed.

## Supplementary Information

Below is the link to the electronic supplementary material.

Supplementary file 1 (pdf 57 KB)

## Data Availability

Data are available through the Edinburgh DataShare repository (10.7488/ds/8031).

## Appendix A: Derivation of the governing equations

The physics of a body settling due to gravity in an incompressible Newtonian fluid can be described by the continuity and momentum equations for incompressible flows and Newtonian fluids, and by Euler’s two equations of motion. The former are

A.1 $$\begin{aligned} \hat{\nabla }\cdot \hat{\varvec{u}} = 0, \end{aligned}$$

A.2 $$\begin{aligned} \frac{\partial \hat{\varvec{u}}}{\partial {\hat{t}}} + (\hat{\varvec{u}}\cdot \hat{\nabla })\hat{\varvec{u}} = \frac{\hat{\nabla }{\hat{p}}}{\hat{\rho }_\text {f}} + \hat{\nu }\hat{\nabla }^{2}\hat{\varvec{u}}, \end{aligned}$$

where $\hat{\varvec{u}}$ is the flow velocity vector; *t* is time; $\nu$ is the fluid kinematic viscosity; and *p* is the kinematic pressure net of the hydrostatic pressure due to the gravitational force. The physical pressure is

A.3 $$\begin{aligned} \hat{p}_\text {ph}=\hat{p}+\hat{\rho }_\text {f}{\hat{g}} \hat{\eta }, \end{aligned}$$

where $\hat{\eta }$ is a vertical coordinate in the direction of $\hat{\varvec{g}}$, with origin where the hydrostatic pressure is zero. Notably, the flow field $\hat{\varvec{u}}(\hat{\varvec{x}})$ is independent of the gravitational force. In fact, a lemma of Archimedes’ principle is that the pressure force due to a uniform pressure gradient results in a buoyancy force equal in magnitude and opposite in sign to the body force that has generated the pressure gradient. Therefore, the forces on the body remain unchanged whether one considers both the gravitational force and the hydrostatic pressure gradient or neither. (The lay reader may be persuaded by considering the flow field around a body whose kinematics is prescribed: the flow field is independent of the orientation of the experiment relative to gravity.)

Here, the kinematics is provided by Euler’s equations, which, instead, depend on the magnitude and orientation of the gravitational force. Euler’s first and second laws of motion for a rigid body describe the conservation of linear and angular momentum, respectively. The two laws of motion are

A.4 $$\begin{aligned} & \hat{m}\dot{\hat{\varvec{u}}}_\text {b} =\hat{\varvec{F}}+\hat{m}\hat{g}^*\varvec{\gamma }, \end{aligned}$$

A.5 $$\begin{aligned} & \quad \hat{\mathbb {I}}_\text {o} \dot{\hat{\varvec{\omega }}}_\text {b} +\hat{\varvec{\omega }}_\text {b} \times \hat{\mathbb {I}}_\text {o} \hat{\varvec{\omega }}_\text {b} =\hat{\varvec{T}}_\text {o} +{\hat{e}}\varvec{r} \times \hat{m}(\hat{g}-\hat{g}^*)\varvec{\gamma }, \end{aligned}$$

where $\hat{m}$ is the mass of the rigid body; $\hat{\varvec{u}}_\text {b}$ is the velocity of the centre of gravity of the rigid body as observed by an arbitrary inertial frame P$(\hat{x},\hat{y},\hat{z})$ such as Earth-fixed; $\hat{\mathbb {I}}_\text {o}$ is the inertia tensor with respect to a non-inertial body-fixed frame centred at the centre of gravity O$(\tilde{x},\tilde{y},\tilde{z})$; $\hat{\varvec{F}}$ is the fluid force exerted on the body; $\hat{\varvec{\omega }}_\text {b}$ is the angular velocity of the solid body with respect to P; $\hat{\varvec{T}}_\text {o}$ is the fluid torque about O; $\varvec{\gamma }$ is a unit vector in the direction of $\hat{\varvec{g}}$; *e* is the Euclidean distance between the centres of gravity and buoyancy; and $\varvec{r}$ is a unit vector from the centre of gravity to the centre of buoyancy.

On the left-hand side of equations A.4 to A.5 are the rates of change of the linear and angular momentum of the solid body, respectively. Equation A.5 is expressed in a non-inertial frame of reference O$(\tilde{x},\tilde{y},\tilde{z})$ centred on the centre of gravity of the rigid body and co-rotating with it. The last term on the right-hand side of equation A.4 is the reduced gravitational force, which is the difference between the gravitational force and the buoyancy force. The last term on the right-hand side of equation A.5 is the moment due to the buoyancy force, which is the difference between the gravitational force and the reduced gravitational force.

We define non-dimensional quantities using the fluid density $\hat{\rho }_\text {f}$, the reference length ${\hat{l}}$ and the gravitational velocity $\hat{u}_g$ as reference parameters. For example, we define $x \equiv {\hat{x}} / {\hat{l}}$, $u \equiv {\hat{u}} / \hat{u}_g$, $p \equiv \hat{p} / (\hat{\rho }_\text {f} \hat{u}^2_g)$, $F \equiv {\hat{F}} / (\hat{\rho }_\text {f} \hat{u}^2_g \hat{l}^2)$, etc. Therefore, substituting ${\hat{x}}$ with $x \hat{l}$, ${\hat{u}}$ with $u \hat{u}_g$, ${\hat{p}}$ with $p \hat{\rho }_\text {f} \hat{u}^2_g$, ${\hat{F}}$ with $F \hat{\rho }_\text {f} \hat{u}^2_g \hat{l}^2$ and so forth, into equation A.1, A.2, A.4 and A.5, we find equation 2.1, 2.2, 2.3 and 2.4.

Notably, the magnitude of the non-dimensional reduced gravitational force is $mg^*=1$. In fact, from the definition of $\hat{u}_g$, one observes that ${\hat{m}} {\hat{g}}^* = \hat{u}^2_g \hat{\rho }_\text {f} \hat{l}^2$, whose non-dimensional form is one. Therefore, the last term on the right-hand side of equation 2.3 is $mg^*\varvec{\gamma }=\varvec{\gamma }$. Conversely, the magnitude of the non-dimensional buoyancy force can be written as a function of the gravitational force as $mg/\rho$, where $\rho$ is the solid-to-fluid density ratio. Alternatively, it can be written as a difference between the gravitational force and the reduced gravitational force as $m(g-g^*)$ consistently with equation A.5. Substituting $g=g^*/(1-1/\rho )$ (from the definition of $g^*$ in Sect. 2.1) and $mg^*=1$ into one of these two forms of the magnitude of the non-dimensional buoyancy force, one finds $1/(\rho -1)$. Therefore, the last term on the right-hand side of equation 2.4 is $e\varvec{r}\times \varvec{\gamma }/(\rho -1).$

## List of symbols

$e$: Euclidean distance between the centres of gravity and buoyancy
$$\textrm{Ga}$$: Galilei number
G: Origin of the non-inertial horizontally accelerating frame of reference G$(x',y',z')$
$$G_\text {R}$$: Gust ratio
$H$: Height of the wind tunnel test section
$l$: Characteristic length of the body
$m$: Mass of the body
O: Origin of the non-inertial body-fixed frame of reference O$(\tilde{x},\tilde{y},\tilde{z})$
$p$: Pressure net of the hydrostatic pressure due to $\varvec{g}$
$p'$: Pressure net of the hydrostatic pressure due to $\varvec{g}$ and $\varvec{\dot{u}}_\text {G}$
$$p_\text {ph}$$: Physical pressure
P: Origin of the inertial frame of reference P(*x*, *y*, *z*)
$$\textrm{Re}$$: Reynolds number
$$T_{u,v}$$: Turbulence intensity
$u$: Velocity in the *x* direction, which is streamwise and vertical
$v$: Velocity in the *y* direction, which is streamnormal and horizontal
$v_\text {G}$: Horizontal speed of the wind tunnel
$$t$$: Time
$u_\textrm{g}$: Gravitational velocity of the body
$u_{\text {t}}$: Terminal velocity of the body
$W$: Width of the wind tunnel test section
$$x$$: Streamwise coordinate
$$y$$: Streamnormal coordinate
$\chi$: Mass geometric factor
$\chi _{i,j}$: Inertia geometric factor
$\epsilon$: Depth coordinate for the hydrostatic pressure due to $\varvec{\dot{u}}_\text {G}$
$\eta$: Depth coordinate for the hydrostatic pressure due to $\varvec{g}$
$\nu$: Fluid kinematic viscosity
$\rho$: Solid-to-fluid density ratio
$\rho _\text {b}$: Density of the body
$\rho _\text {f}$: Density of the fluid
$\theta$: Angular position of the body
$$\varvec{b}$$: Buoyancy force
$\varvec{F}$: Fluid force exerted on the body
$$\varvec{g}$$: Gravitational acceleration
$\varvec{g}^*$: Reduced gravity acceleration, i.e. net of its specific buoyancy force
$\varvec{r}$: Unit vector from the centre of buoyancy to the centre of gravity of the body
$\varvec{T}_\text {o}$: Fluid torque exerted on the body about its centre of gravity
$\varvec{u}$: Velocity with respect to the frame P, $\varvec{u}\equiv (u,v,w)$
$\varvec{u}'$: Velocity with respect to the frame G
$\varvec{u}_\text {b}$: Velocity of the body’s centre of gravity with respect to the frame P
$\varvec{u}'_\text {b}$: Velocity of the body’s centre of gravity with respect to the frame G
$\varvec{u}_\text {G}$: Velocity of the frame G with respect to the frame P and gust velocity
$\varvec{\dot{u}}^*_\text {G}$: Reduced gust acceleration, i.e. net of its specific buoyancy force
$\varvec{x}$: Position vector with respect to the frame P, $\varvec{x}\equiv (x,y,z)$
$\varvec{x'}$: Position vector with respect to the frame G, $\varvec{x'}\equiv (x',y',z')$
$\varvec{\gamma }$: Unit vector in the direction of the gravitational acceleration
$\varvec{\omega }_\text {b}$: Angular velocity of the body
$\varvec{\omega }_\text {G}$: Angular velocity of the frame G with respect to the frame P
$\mathbb {I}_\text {o}$: Inertia tensor with respect to the frame O
MAV: Microaerial vehicle
PIV: Particle Image Velocimetry
