# Supplementary material for: An accelerating wind tunnel for testing untethered bodies in transverse gusts
Source: Exp Fluids. 2025 Oct 25;66(11):205. doi: 10.1007/s00348-025-04135-5 (PMC12553603; doi:10.1007/s00348-025-04135-5)
Supplement: Supplementary file 1 — (pdf 57 KB) [file 348_2025_4135_MOESM1_ESM.pdf]

An Accelerating Wind Tunnel for Testing Untethered Bodies in Transverse Gusts: Supplementary Material

by I. M. Viola, A. Potnis, S. Bhattacharyya, E. J. Williams, D. Halley and D. Murphy

Mie-scattering and Particle image velocimetry

A 2.5 W diode-pumped continuous wave laser (Medialas DPGL 2500) with a wavelength of 532 nm, coupled with sheet-making optics, was used to create a < 1 mm thick vertical laser sheet positioned in the planes shown in figure . The flow, seeded with DEHS. (di-ethyl hexyl sebacate) droplets, was illuminated by the laser sheet. A high-speed Phantom Veo 640 S camera, equipped with a Nikon 200 mm f/4 lens and placed orthogonal to the laser plane, captured Mie scattering images at 1000 fps and with a resolution of 2560 pixels $\times$ 1600 pixels providing a spatial resolution of ≈ 0.044 mm/pixel.

The Mie-scattering images were processed using LaVision's Davis 10 software to obtain the velocity field via PIV. A multi-pass linear window was used for cross-correlation with four final passes; the interrogation window was 64 pixel $\times$ 64 pixel with a 50% overlap. Time-averaged velocity fields were merged in overlapping regions to generate the data illustrated in figure 4.

Methodology for Image Analysis and Object Tracking

High-speed images were captured using a Photron SA-6 camera, with resolution of 1920 pixels $\times$ 1440 pixels at 750 fps, and later processed with the open-source image analysis software Fiji.

Circular markers, measuring 6 ± 0.5 mm were stuck to the back panel of the tunnel. in diameter, were attached to the back panel of the tunnel. The tunnel was backlit to produce shadowgraphy images of the markers, which moved in tandem with the tunnel. High-speed shadowgraphy imaging captured the motion of the tunnel and its attached markers. These images were subsequently analysed in Fiji, following a series of steps to enhance the visibility and track the movement of the markers. First, the brightness and contrast were adjusted to achieve a clear, high-contrast image. Background subtraction followed, using a sliding paraboloid with a diameter of 10 pixels. The images were then binarised, with thresholding applied to preserve the marker shapes while eliminating background artifacts. Individual frames were analysed to locate the markers, employing the 'analyse particles' function with filters to exclude noise and spurious particles. The centroids of the detected markers were tracked to capture the tunnel's movement, with multiple markers tracked to provide a continuous, robust record of tunnel displacement.

A similar approach was applied to track the natural flyer, focusing on recording the height of the seed's bottom tip by identifying the bounding box surrounding the flyer.

To track both the tunnel and the flyer simultaneously, the images were appropriately cropped and filtered, enabling differentiation between the two objects while using 'analyse particles'.
